# The role of probiotics in the immune response and intestinal microbiota of children with celiac disease: a systematic review

**DOI:** 10.1590/1984-0462/2022/40/2020447

**Published:** 2021-09-01

**Authors:** Camila Fernanda Jedwab, Bruna Cardoso de Mattos Boccalini Roston, Ana Beatriz Ferreira de Souza Toge, Isadora Fagundes Echeverria, Guilherme Ojea Gomes Tavares, Matheus Alves Alvares, Vera Esteves Vagnozzi Rullo, Marcella Rocha Machado de Oliveira

**Affiliations:** a Centro Universitário Lusíada, Santos, SP, Brazil.; b Universidade Federal de São Paulo, São Paulo, SP, Brazil.

**Keywords:** Celiac disease, Probiotics, Gastrointestinal microbiome, Diet, gluten free, Bifidobacterium breve, Bifidobacterium longum, Doença celíaca, Probióticos, Microbioma gastrointestinal, Dieta livre de glúten, Bifidobacterium breve, Bifidobacterium longum

## Abstract

**Objective:**

To evaluate changes in peripheral immunological response (decrease in blood proinflammatory cytokines) and fecal microbiota (especially *Bacteroidetes* and *Firmicutes*) after administration of probiotics in children with celiac disease on a gluten-free diet.

**Data source:**

The databases MEDLINE, LILACS, Springer and SciELO were used for this review, with the descriptors “celiac disease AND probiotics”. At the end of the search, 168 articles were retrieved, four of which were included in the final qualitative synthesis, having as inclusion criteria randomized clinical trials and pediatric population (1–19 years) and, as exclusion criteria, interventions other than probiotics, studies with patients with other diseases associated with celiac disease, or patients who did not meet the diagnostic criteria. All elected studies were published until September 2020, without language restriction, with patients receiving strains of Bifidobacterium breve or B. longum and on a gluten-free diet.

**Data synthesis:**

The studies show that the administration of probiotics along with a gluten-free diet, can approximate the fecal microbiota of celiac patients to typical conditions of healthy individuals, by restoring the abundance of some microbial communities that characterize the typical physiological condition. In addition, the administration of probiotics can reduce serum proinflammatory cytokines (mainly TNF-alpha).

**Conclusions:**

Despite the positive correlation between probiotics and fecal microbiota/serological markers in pediatric patients with celiac disease, we emphasize the need for future multicentric studies that should include a larger number of patients and a longer follow up period.%

## RESUMO

**Objetivo:**

Avaliar mudanças na resposta imunológica (diminuição de citocinas pró-inflamatórias no sangue) e na microbiota fecal (principalmente Bacteroidetes e Firmicutes) após a administração de probióticos em crianças com doença celíaca que seguiam uma dieta livre de glúten.

**Fontes de dados:**

Foram utilizadas para esta revisão as bases de dados Medical Literature Analysis and Retrieval System Online (MEDLINE), Literatura Latino-Americana e do Caribe em Ciências da Saúde (LILACS), Springer e Scientific Electronic Library Online (SciELO), com os descritores “celiac disease AND probiotics”. No fim da busca, foram recuperados 168 artigos, dos quais quatro foram incluídos na síntese qualitativa final. Os critérios de inclusão foram ensaios clínicos randomizados e população pediátrica (1–19 anos) e os critérios de exclusão, intervenções que não fossem com probióticos, estudos com pacientes portadores de outras doenças associadas à doença celíaca, ou que não preenchessem critérios para o diagnóstico. Todos os estudos eleitos foram publicados até setembro de 2020, sem restrição de idioma, com pacientes recebendo cepas de Bifidobacterium breve ou B. longum e em uma dieta livre de glúten.

**Síntese dos dados:**

Os estudos demonstram que a administração de probióticos associada a uma dieta livre de glúten pode aproximar a microbiota fecal desses pacientes às condições típicas de um indivíduo saudável ao restaurar a abundância de algumas comunidades microbianas que caracterizam uma condição tipicamente fisiológica. Além disso, a administração de probióticos pode reduzir as citocinas pró-inflamatórias (principalmente TNF-alfa).

**Conclusões:**

Apesar da correlação positiva entre probióticos e microbiota fecal/marcadores sorológicos nos pacientes pediátricos portadores de doença celíaca, ressalta-se a necessidade de estudos multicêntricos futuros que abranjam um maior número de pacientes e maior tempo de acompanhamento.%

## INTRODUCTION

Celiac disease (CD) is a chronic inflammatory condition of the gastrointestinal tract, which primarily affects the small intestine, causing villous atrophy and malabsorption of nutrients.^1^ It occurs in genetically susceptible individuals, who, in response to environmental triggers, develop an immune response secondary to ingestion of gluten.^2^ Patients can present different degrees of intestinal inflammation, from intraepithelial lymphocytosis to severe subepithelial infiltration, resulting in villous atrophy and crypt hyperplasia. The clinical and laboratory presentation of the disease can vary from asymptomatic^3^ to the classic malabsorptive or disabsorptive picture,^4^ which is more commonly found in children than adults.^5^ Its diagnosis is based on the recognition of symptoms, serological screening and biopsies of the small intestine.^4^

In recent years, CD has been recognized as one of the most common chronic disorders in children, with estimated prevalence of 1%.^6^ Positive family history, genetic problems and other autoimmune conditions are considered risk factors for the development of the disease.^3^

Currently, the intestinal microbiota and its potential relationship with inflammatory and autoimmune diseases have been widely discussed in the literature. The three phyla of bacteria that make up most of the components of this microbiome are *Firmicutes*, *Bacteroidetes* and *Actinobacteria*.^7^ In general, most duodenal and fecal biopsies of patients with CD compared to healthy individuals show a certain dysbiosis, with a decrease in *Lactobacillus* spp., *Enterococci*, *Firmicutes* and *Bifidobacterium* and an increase in the number of Gram negative bacteria, Bacteroidetes and *Staphylococcus*.^8^ This imbalance has been proven particularly important in the pathogenesis of CD, raising the interest in probiotics as therapeutic targets.

Probiotics are live microorganisms that, when consumed in adequate amounts, can have preservative effects on the intestinal epithelial barrier and immunomodulatory effects (inhibition of pro-inflammatory cytokines such as IFN-gamma, TNF-alpha, IL-2).^7^ In addition, they can provide apoptosis of epithelial cells and adhesion of pathogenic bacteria in the intestines.^9^

It is known that the only treatment currently available for CD is a strictly gluten-free diet, which promotes almost complete resolution of symptoms and healing of the mucosa; however, different studies have shown that patients with CD have problems adhering entirely to this type of diet, which leads to numerous complications of the disease.^10^ ^–^ ^12^ Thus, several therapeutic alternatives have emerged, but so far no medication is widely available on the market. The association of a gluten-free diet with probiotics has been proven effective *in-vitro* and in animals.^13^

The current literature is still controversial on the subject. Therefore, this study aims to provide an updated review regarding the use of probiotics, mainly *Bifidobacterium breve* and *B. longum*, in the immunological parameters and intestinal microbiota of pediatric patients with CD.

## METHOD

This systematic review of randomized controlled trials (RCTs) sought to assess the effects of probiotics on the fecal microbiota and on serological markers of pediatric patients diagnosed with CD. The articles were selected according to the recommendations of the Preferred Reporting Items for Systematic Reviews and Meta-Analyses (The PRISMA Statement), responsible for coordinating the processes to conduct meta-analyses and systematic reviews.^14^

For the selection of papers, a systematic search of the literature was carried out in the databases Medical Literature Analysis and Retrieval System Online (MEDLINE) (via PubMed), Latin American and Caribbean Literature in Health Sciences (LILACS) (via Virtual Library in Health), Springer and Scientific Electronic Library Online (SciELO). The following terms were used to search the databases: MEDLINE – (probiotic[MeSH Terms]) OR (probiotics[MeSH Terms]) AND (celiac disease[MeSH Terms]); LILACS – tw:(celiac disease)) AND (tw:(probiotic)); Springer – “celiac disease” AND (probiotics OR probiotic); SciELO – (probiótico) AND (doença celíaca). The searches were made out between August and September 2020, without language restrictions.

The inclusion criteria were only RCTs and studies conducted with the pediatric population (from 0 to 19 incomplete years old). The exclusion criteria were observational studies, studies with non-pediatric population, literature reviews, duplicate studies, interventions that were not with probiotics, studies with patients with other diseases associated with CD, patients with non-celiac gluten sensitivity and patients with genetic predisposition to CD, but without confirmed diagnosis (according to the criteria of the European Society for Pediatric Gastroenterology Hepatology and Nutrition, ESPGHAN).

**Figure 1:**
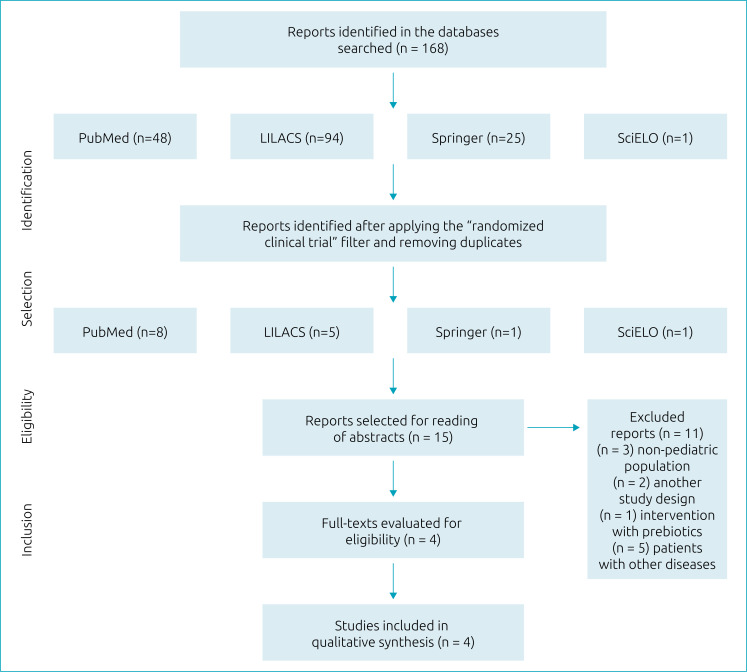
Flowchart of inclusion of studies in the analysis.

After a systematic review of the literature based on these databases, articles that met the inclusion criteria were selected. A total of 168 articles were reached: 48 via MEDLINE, 94 via LILACS, 25 via Springer and one via SciELO. After methodological screening, four studies were eligible for this systematic review. Figure 1 describes the steps of papers’ selection.

## RESULTS

The four papers selected for this systematic review are RCTs with a control group that received only placebo, and an intervention group that received probiotics. The object of study was the pediatric population (minimum 1 and maximum 19 years) with diagnosis of CD confirmed by the ESPGHAN criteria,^15^ totaling 165 participants. The characteristics of each study are detailed in Table 1.

**Table 1:** Characteristics of the randomized controlled trials selected.

Author, year, country	Cohort age range (years)	Probiotic	Intervention group (n)	Placebo group (n)	Follow-up time
Quagliariello et al., 2016, Slovenia^17^	1-19	*B. breve* B632 (DSM 24706) and BR03 (DSM 16604)	20	20	3 months
Klemenak et al., 2015, Slovenia^18^	1-19	*B. breve* B632 and BR03	24	25	3 months
Olivares et al., 2014, Spain^19^	2-17	*Bifidobacterium longum* CECT 7347	18	18	3 months
Primec et al., 2018, Slovenia^20^	1-19	*B. breve* B632 and BR03	20	20	3 months

The clinical trials were individually assessed for methodological quality (risk of bias) based on the Cochrane risk of bias assessment tool (Cochrane Risk of Bias Tool)^16^ and are further detailed in Table 2.

**Table 2:** Cochrane Collaboration Tool for the assessment of risk of bias.^16^

Studies	Quagliariello et al.^17^	Klemenak et al.^18^	Olivares et al.^19^	Primec et al.^20^
Random sequence generation	B	A	A	B
Allocation concealment	A	A	A	B
Blinding of participants and professionals	A	A	A	A
Blinding of outcome evaluators	A	B	A	A
Incomplete outcomes	B	A	A	B
Report of selective outcome	B	A	B	A
Other sources of bias	B	B	B	B

A: low risk of bias; B: uncertain risk of bias; C: high risk of bias.

Quagliariello et al.^17^ conducted a study with 40 pediatric patients aged 1 to 19 years old who were on a gluten-free diet and were positive for serological CD and small bowel biopsy (according to the ESPGHAN^14^ criteria for CD diagnosis). Patients with any other acute or chronic disease, on permanent medications and who had taken antibiotics within one month before the start of the study were excluded. Participants were randomized to receive either *B. breve* B632 (DSM 24706) and BR03 (DSM 16604) strains in a ratio of 1:1 (n=20) or placebo (n=20) for three months. The researchers analyzed microbial DNA (using a polymerase chain reaction – real-time PCR) extracted from the feces of patients with CD before and after treatment with probiotics or placebo. In addition, they analyzed the feces of a control group with 16 healthy patients (with no comorbidities).

DNA sequencing showed significant presence of six phyla of bacteria: Firmicutes, Bacteroidetes, Proteobacteria, Actinobacteria, Verrucomicrobia and Euryarchaeota. An increase in Actinobacteria and the restoration of the physiological proportion of Firmicutes/Bacteroidetes in the intervention group after three months of treatment were seen, leading to the belief that the administration of *B. breve* strains associated with a gluten-free diet can bring the fecal microbiota of celiac patients closer to the typical conditions of a healthy individual.

In the study by Klemenak et al.,^18^ 49 children aged 1 to 19 years and diagnosed with CD were selected on a gluten-free diet. The exclusion and inclusion criteria were the same as those adopted in the study conducted by Quagliariello et al. This population was randomized to receive, if allocated to the intervention group, *B. breve* strains B623 and BR03 daily for three months (n=24), and placebo if allocated to the control group, in the same period (n=25). In addition, 18 healthy children were assessed as a control group. The primary outcome measure was the assessment of change in serum levels of cytokines TNF-α and IL-10 after the administration of the aforementioned probiotics, aiming to decrease the intestinal pro-inflammatory environment of celiac patients and, therefore, the incidence of complications of the disease. The study concluded that TNF-alpha levels decreased significantly with the administration of *B. breve* probiotics after three months (p=0.02). However, three months after the end of the intervention (follow-up), the levels of TNF-alpha rose again. IL-10 levels were below the detection level (5pg/mL) and, therefore, were not quantified.

Olivares et al.^19^ conducted a study with patients in the pediatric age group (2 to 17 years), in which 36 children with newly diagnosed CD according to the ESPGHAN criteria were included. Exclusion criteria were patients simultaneously involved in other clinical studies, treatment with antibiotics 30 days before the start of the study, and/or food allergies and other pathologies. Children were randomized into an intervention group and a control group. In the former, they received *Bifidobacterium longum* CECT 7347 (n=18) and, in the latter, placebo (n=18) for three months, in addition to a gluten-free diet. The primary outcome of this study was to assess whether oral intake of *B. longum* CECT 7347 would improve the effectiveness of the gluten-free diet. The secondary outcome included the change in anthropometric (weight and height) and immunological (cytokines and lymphocytic phenotyping) parameters of the patients, in addition to the evaluation of the composition of the intestinal microbiota after three months of intervention by analyzing the secretory IgA (sIgA) and the DNA from different bacteria in the stool. The authors concluded that the administration of this probiotic associated with a gluten-free diet leads to changes in the intestinal microbiota, mainly the reduction of levels of bacteria with potentially pro-inflammatory effects, such as *B. fragilis*, and the reduction of levels of sIgA. In addition, it leads to a reduction in peripheral CD3+ T lymphocytes and TNF-alpha, which can contribute to the recovery of immune homeostasis in pediatric patients with CD.

The study by Primec et al.^20^ evaluated the effect of probiotics in 40 patients (aged 1 to 19 years) with a previous diagnosis of CD according to the ESPGHAN criteria, on a gluten-free diet, including those in the intervention group (n=20), who received strains of *Bifidobacterium breve* B632 and BR03 for three months, while the other subjects received placebo for the same period. Sixteen healthy children on a gluten-free diet made up the control group. Patients with any other acute or chronic disease, on permanent medications and who had taken antibiotics up to one month before the study were excluded from the control group. The primary outcome was the assessment of the influence of such probiotics on the fecal microbiota by the extraction of bacterial DNA, in addition to short-chain fatty acids (SCFA) and serum TNF-alpha. The study concluded that Verrucomicrobia, Parcubacteria and some other unknown phyla of Bacteria and Archaea may be involved in the increased production of TNF-alpha in patients with CD. Furthermore, the presence of Proteobacteria pointed out a possible relationship with the increase in fecal SCFA in this disease.

Regarding TNF-alpha levels, Klemenak et al. and Olivares et al. reported a drop in levels of this cytokine after three months of probiotic administration (p=0.020 and p=0.067, respectively). Olivares et al. also showed a decrease in CD3+ T lymphocytes (p=0.004), suggesting that Bifidobacterium could contribute to the reduction of the patients’ inflammatory status when associated with a gluten-free diet.

Primec et al. reported, in healthy children, a strong negative correlation between TNF-alpha and Firmicutes (r=0.660, p=0.010). This study also pointed a negative association of TNF levels with Firmicutes (r=0.468, p=0.038) after administration of *B. breve* BR03 and *B. breve* B632 for three months, which corroborates the studies by Klemenak et al. and Quagliariello et al., showing a decrease in this cytokine and reestablishing the Firmicutes/Bacteroidetes ratio, respectively. In the placebo group, TNF-a had a negative correlation (r=0.507, p=0.032) with Bacteroidetes after the same follow-up period. This suggests that this probiotic may bring the intestinal microbiota of a celiac patient closer to that of a healthy person.

The study by Quagliariello et al. reported, in the metagenomic analysis, that the levels of Firmicutes in the feces were significantly lower in the placebo group (40–50%) compared with the probiotic group (50–60%) after three months of intervention (p<0.01). Bacteroidetes levels, on the other hand, did not undergo statistically significant changes as a result of treatment with probiotics (p>0.01). This differs from the analysis made by Olivares et al., in which the levels of the *B. fragilis* group increased significantly in the placebo group and decreased in the probiotic group (p=0.020).

Regarding the levels of secretory IgA in feces, Olivares et al. reported a significant reduction of this parameter in the *B. longum* CECT 7347 group compared to the placebo (p = 0.011) in three months of follow-up.

Primec et al., in turn, found a positive correlation, at T0 in celiac patients, between Proteobacteria with acetic acid and propionic acid (r=0.452, p=0.004 and r=0.331, p=0.045, respectively), resulting in a positive correlation between these bacteria and total SFCA count (r=0.380, p=0.017). At the end of the intervention, patients given probiotics showed a negative association (r=0.502, p=0.024; r=0.498, p=0.026 and r=0.524, p=0.018) between acetic acid and Verrucomicrobia, unclassified groups of Bacteria and Euryarchaeota, respectively. This suggests that the modulation of SFCA production may play an important role in restoring the intestinal microbiota.

Tables 3 and 4 show the statistical results of the studies.

**Table 3:** Primary outcome: assessment of immunological parameters.

Study	Parameter	PR T0	PR T1	PL T0	PL T1	p-value †	Control group
Klemenak et al.^18^ *	TNF-alpha (pg/ml)	14.8±6.4	12.0±3.6	10.6±3.6	NS	0.020	13.2±4
Olivares et al.^19^ **	TNF-alpha (pg/ml)	1803.3±188.0	1443.9±153.2	1669.6±338.5	1515.2±254.3	0.067	-
	T lymphocytes (%)	70.0±2.2	63.4±2	67.0±2.8	70.0±2.2	0.004	-
Primec et al.^20^	Correlation TNF-alpha with intestinal microbiota (negative or positive)	NS	*Firmicutes*, negative (r= 0.468, p= 0.038)	*Verrucomicrobia*, positive (r=0.780, p<0.001); *Parcubacteria*, negative (r= 0.590, p=0.010)	*Verrucomicrobia,* positive (r= 0.495, p=0.037); *Parcubacteria:* NS; *Bacteroidetes*, negative (r= 0.507, p=0.032)	- ††	*Firmicutes*, negative (r=0.660, p=0.010); *Euryarchaeota*, negative (r=0.654, p= 0.011).

* Klemenak used medians and interquartile ranges;

** Olivares used mean and standard deviation; PR Probiotic; PL placebo; NS non-significant; T0 zero time; T1 after three months; r Spearman's correlation coefficient (which can be positive or negative);

† p-value between groups at the end of the intervention;

†† Analysis between groups not performed.

**Table 4:** Secondary outcome: alteration in fecal microbiota.

Study	Parameter	PR T0	PR T1	PL T0	PL T1	p-value †	Control group
Primec et al^.20^	Correlation SCFA-fecal microbiota	NS	Acetic acid, negative correlation with Verrucomicrobia (r=0.502. p=0.024). unclassified groups of Bacteria (r=0.498. p=0.026). Euryarchaeota (r=0.524. p=0.018) and Synergistetes (r=0.587. p=0.006)	Proteobacteria, positive correlation with total SCFA (r=0.380. p=0.017); Euryarchaeota, positive correlation with acetic acid (r=0.351. p=0.029)	Proteobacteria, positive correlation with acetic acid, total propionic and SCFA (r=0.574. p=0.010 e r=0.505. p=0.027); Acetic acid, negative correlation with unclassified groups of bacteria (r=0.521. p=0.022); Propionic acid, negative correlation with Synergistetes (r=0.471. p=0.042); Butyric acid, positive correlation with Proteobacteria (r= 0.498. p=0.030)	- ††	NS
Olivares et al.^19^ *	Secretory IgA (mg/ml)	77.0±4.9	60.8±7.2	67.1±5.7	84.1±6	0.011	-
	fecal microbiota (B. fragilis) (Log CFU/g of feces)	9.4±0.2	9.3±0.2	9.1±0.2	9.6±0.2	0.020	-
Quagliariello et al.^17^	fecal microbiota (Firmicutes %)	40–50%	50-60%	40–50%	40–50%	<0.01	60–70%
	fecal microbiota (Bacteroidetes %)	30–40%	30–40%	20–30%	30–40%	NS	10–20%

* Olivares used mean and standard deviation; PR Probiotic; PL placebo; NS non-significant; T0 zero time; T1 after three months; r Spearman's correlation coefficient;

† p-value between groups at the end of the intervention;

†† Analysis between groups not performed; SCFAs short-chain fatty acids.

## DISCUSSION

Probiotics have recently been used in several clinical trials as an additional treatment combined with a gluten-free diet in patients with CD, mainly *Bifidobacterium* and *Lactobacillus*. This is due to the fact that these patients have a change in the composition of the intestinal microbiota, marked by a reduction in beneficial bacteria, such as *Bifidobacteria* and *Lactobacillus*; and increased levels of potentially pathogenic microorganisms, such as Proteobacteria, Bacteroidetes and Actinobacteria.^21^ ^–^ ^23^ Patients with CD also have a disproportion of Firmicutes/Bacteroidetes, and this ratio is not completely restored only with a gluten-free diet.^24^

The literature indicates a strong tendency to introduce strains of *Bifidobacterium breve* and *longum* in the diet. These probiotics act as potent modulators of the peripheral immune response, reducing pro-inflammatory cytokines (TNF-alpha), inducing the production of anti-inflammatory cytokines (IL-10) by the Th1 immune response, and preventing inflammation of the intestinal mucosa.^25^ ^,^ ^26^ In addition, they can restore the disproportion between Firmicutes/Bacteroidetes in individuals with CD, as observed by Quagliariello et al.

In the studies analyzed here, homogeneity is seen in terms of the type of intervention (*B. breve* B632 and BR03 in the ratio of 1:1), dose used (10^9^ CFU/g, colony-forming units), follow-up time (three months), age range of the cohort (from 1 to 19 years), presence of a control group with healthy children and location of the study (Slovenia) in the works by Klemenak et al., Primec et al. and Quagliariello et al. Only Olivares differs by the use of *B. longum* CECT 7347 in 10^9^ CFU/g, by the age range of 2 to 17 years, the absence of a control group, and by being conducted in Spain.

With regard to the limitations of the studies, the reduced number of articles analyzed in this review, which implies a reduced cohort in each one, is a factor to be considered. The fact that the works were not conducted in multiple centers and only in Europe also limits the applicability of probiotic therapy. Another limiting factor is the time and rate of adherence to the gluten-free diet.

In the study by Quagliariello et al., the group that received probiotics was on a gluten-free diet for 5.4±3.4 years, with 90% adherence, while the placebo group was on this diet for 7.2±5 years and had an adherence of 80%. Klemenak et al. had in the probiotic group children who had followed this diet for 5.6±3.7 years, with 80% adherence, and, in the placebo group, for 7.1±5.5 years with 91% adherence. Primec et al. did not detail this information, they only mentioned the follow-up of a gluten-free diet among all participants for 0.5±15 years. Finally, Olivares et al. assessed children newly diagnosed with CD and, therefore, who started on the diet at the beginning of the intervention. Such discrepancies between studies may imply biased results, since it is known that strict adherence to the gluten-free diet is essential for the treatment of the disease.^24^

In addition to the papers evaluated in this review, other authors have reported, both in clinical trials and in observational studies, benefits of the administration of other probiotics. Hakansson et al. assessed children with genetic susceptibility to CD and not on a gluten-free diet who received L. *plantarum* HEAL9 and L. *paracasei* 8700:2 or placebo for six months. In the observed result, interestingly, the difference in most of the lymphocyte subtypes found in the placebo group was similar to that found in patients with active CD, indicating a disease progression that was not observed in the probiotic group.^27^

An observational cohort study conducted in Brazil prospectively analyzed children with CD and healthy children who ate yogurt daily with a mixture of probiotics for 30 days and demonstrated a significant increase in *Bifidobacteria* in the stools of celiac patients, but not in healthy participants, after the intervention.^28^ This goes against the findings of Olivares et al., whose research reported the intervention group without a statistically significant increase (p=0.875) in the count of *Bifidobacterium* spp. in the feces, also for the placebo group (p=0.151).

Currently, the only existing treatment for CD is a strictly gluten-free diet for life, according to the latest guidelines of the World Gastroenterology Organization (WGO), 2016.^29^ Despite the fact that new probiotic therapy options have reached the stage of clinical trials, proving to be safe, none of them are widely available for clinical use. In addition, the current literature does not yet include large studies carried out in the pediatric population with CD. Thus, further investigations are needed to confirm the real benefit of these probiotics as a complementary therapy associated with a gluten-free diet for children with CD.
